# *ZBTB38* suppresses prostate cancer cell proliferation and migration via directly promoting *DKK1* expression

**DOI:** 10.1038/s41419-021-04278-3

**Published:** 2021-10-25

**Authors:** Guanxiong Ding, Wei Lu, Qing Zhang, Kai Li, Huihui Zhou, Fei Wang, Chunchun Zhao, Caibin Fan, Jianqing Wang

**Affiliations:** 1 Department of Urology, Huashan Hospital, Fudan University, Shanghai, China; 2 School of Nursing, Suzhou Vocational Health College, Suzhou, China; 3 Department of Urology, The Affiliated Suzhou Hospital of Nanjing Medical University, Suzhou Municipal Hospital, Gusu School, Nanjing Medical University, Nanjing, China; 4 Department of pathology, Affiliated Yuhuangding Hospital of Qingdao University, Qingdao, China

**Keywords:** Tumour-suppressor proteins, Prostate cancer

## Abstract

Prostate cancer is still one of the most common malignancies in men all around the world. The mechanism of how prostate cancer initiates and develops is still not clear. Here in this study, we show that tumor suppressor *ZBTB38* could suppress the migration and proliferation of prostate cancer cells. We find lower *ZBTB38* expression in prostate cancer tissues, which also strongly predicts a poorer prognosis of prostate cancer. *ZBTB38* binds *DKK1* (Dickkopf *WNT* signaling pathway inhibitor 1) locus and promotes *DKK1* expression in prostate cancer cell lines. Consistently, reduction of *DKK1* expression significantly restores *ZBTB38*-mediated suppression of migration and proliferation of prostate cancer cell lines. Mechanistically, we find that *ZBTB38* primarily binds the promoters of target genes, and differentially regulates the expression of 1818 genes. We also identify *PRKDC* (protein kinase, DNA-activated, catalytic subunit) as a *ZBTB38*-interacting protein that could repress the function of *ZBTB38* in suppressing migration and proliferation of prostate cancer cells. Taken together, our results indicate that *ZBTB38* could repress cell migration and proliferation in prostate cancer via promoting *DKK1* expression, and also provide evidence supporting *ZBTB38* as a potential prognosis marker for prostate cancer.

## Introduction

Prostate cancer is still one of the most common causes of cancer-related death in males, particularly in western countries [1]. The incidence of prostate cancer is increasing rapidly all around the world. Although comprehensively studied, the underlying mechanism of the initiation and development of prostate cancer is still not fully understood.

Zinc finger and BTB domain containing 38 (*ZBTB38*, also known as *CIBZ*, *ZNF921m*, or *PPP1R171*) is a new member of the ZBTB family located on chromosome 3q23. ZBTB (zinc finger and BTB domain protein family) family refers to a kind of proteins that contain multiple zinc finger domains at the C-terminus and a BTB domain at the N-terminus [2]. These proteins play important roles in growth, tumorigenesis, cytoskeleton organization, stem cell homeostasis, transcription regulation, hematopoiesis, chromatin remodeling, and protein degradation through synergistic effects [3]. *ZBTB38* gene consists of 8 exons, and its transcription product contains BTB binding domain, CtBr binding domain, and C2H2 zinc finger domain, which has a high degree of homology among primates [4]. *ZBTB38* is a new gene with unclear functions. Previous studies found that it can bind to methylated DNA to inhibit the transcription of insulin-like growth factor 2 (*IGF-II*) [4, 5]. In addition, *ZBTB38* protein can regulate the cleavage and activation of caspase-3 (Caspase-3) through non-DNA methylation mechanisms [6]. Previous large-scale GWAS studies found that rs6763931, an SNP site located in *ZBTB38* gene region, is a prostate cancer risk SNP (11, 12), which indicated the potential role of *ZBTB38* in prostate cancer. Moreover, only one previous research last year showed the preliminary study on the role of *ZBTB38* in the initiation and progression of prostate cancer [7]. However, the roles and mechanisms of *ZBTB38* in prostate cancer are still unclear and need further research.

Dickkopf *WNT* Signaling Pathway Inhibitor 1 (*DKK1*) is a secreted protein with two cysteine-rich domains that could mediate protein–protein interactions, which is a member of the dickkopf family proteins. Previous studies showed that *DKK1* could inhibit β-catenin-dependent Wnt signaling by inhibiting *LRP5/6* interaction with Wnt, and play important roles in embryonic development and bone formation in adults [8–10]. It has been observed that *DKK1* shows abnormal expression in various types of human cancers and may promote or inhibit proliferation and invasion in cancer cell lines [11–14]. The role and expression pattern of *DKK1* in prostate cancer are complicated. Some results indicate that *DKK1* expresses highly in prostate cancer tissues and promotes cancer cells proliferation and migration [15]. However, some researches later showed that *DKK1* expression decreases in metastasis progression in prostate cancer, despite the high expression level in early development, indicating the potential tumor suppressor effect in advanced prostate cancer [16, 17]. Therefore, the role and expression regulating mechanism of *DKK1* in prostate cancer remain to be further studied.

In this study, we studied the function and mechanism of *ZBTB38* in the progression of prostate cancer systematically. We found that overexpression of *ZBTB38* could repress the proliferation and migration of prostate cancer cells, and its expression is reversely correlated with prostate cancer disease progression. Mechanistically, our results indicated that *PRKDC* could interact with *ZBTB38* and repress the function of *ZBTB38*. Knocking down *PRKDC* could promote *DKK1* expression to suppress the malignant progression of prostate cancer cells.

## Results

### Lower *ZBTB38* expression correlated with prostate cancer development

**Fig. 1:**
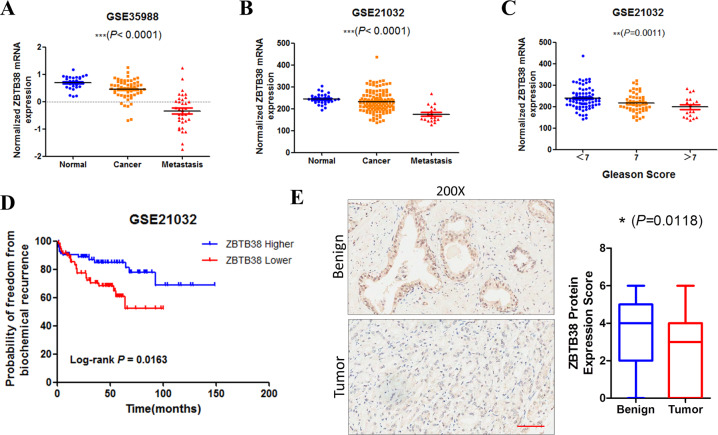
*ZBTB38* expression level correlates with prostate cancer progression and prognosis. **A**, **B** *ZBTB38* mRNA expression levels in prostate cancer samples from GSE35988 (**A**) and GSE21032 (**B**). One-way ANOVA analysis was used. **C** *ZBTB38* mRNA expression levels in prostate cancer samples with different Gleason Score groups in GSE21032. One-way ANOVA analysis was used. **D** *ZBTB38* mRNA level was associated with prostate cancer prognosis. A statistically significant increase in RFS was observed in patients with a higher *ZBTB38* expression group (*P* = 0.0163). **E** Representative IHC images of *ZBTB38* in prostate cancer tissues (*N* = 94 in Benign and cancer group, respectively; Scale bar: 100 μm).(**P < 0.01, ***P < 0.001).

To explore the function of *ZBTB38* in prostate cancer, we first determined the expression level of *ZBTB38* in benign and cancer tissues. Microarray-based expression analyses using GSE35988 and GSE21032 data sets indicated significantly reduced *ZBTB38* expression in primary and metastatic prostate cancer samples (Fig. 1A, B). Moreover, we found that *ZBTB38* downregulation was also related to the progression and prognosis of prostate cancer. In Fig. 1C, we showed that prostate cancer samples with higher Gleason scores showed the lower level of *ZBTB38* mRNA expression level. Patients with a lower *ZBTB38* level (lower than the median value of ZBTB38 expression) showed a much poorer prognosis (Fig. 1D). To confirm the results from online data sets, we examined the expression of *ZBTB38* by immunohistochemistry (IHC) in prostate cancer tissues. As shown in Fig. 1E, the expression of *ZBTB38* is significantly lower in cancer tissues compared with benign prostatic epithelia. Our results above showed the lower expression level of *ZBTB38* in prostate cancer and suggested the potentially important role of *ZBTB38* in disease progression.

### Overexpression of *ZBTB38* suppressed prostate cancer proliferation and migration

**Fig. 2:**
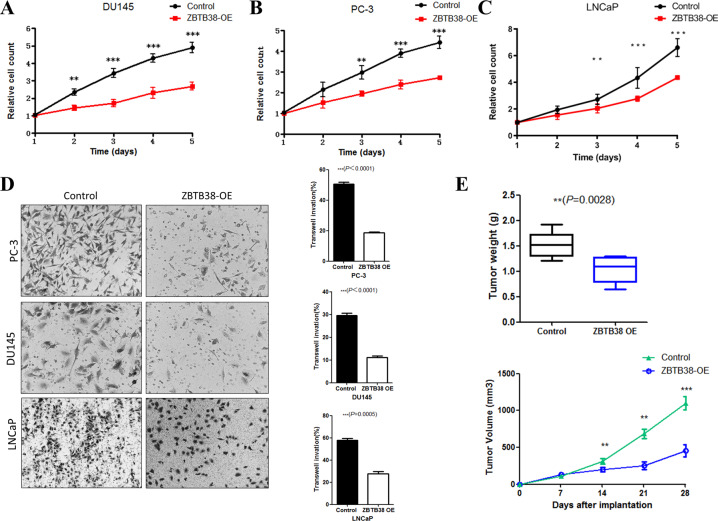
*ZBTB38* suppresses prostate cancer cell proliferation and migration. **A** Cell proliferation was measured at the indicated time points in DU145 cells (**P < 0.01, ***P < 0.001). **B** Cell proliferation was measured at the indicated time points in PC-3 cells (**P < 0.01, ***P < 0.001). **C** Cell proliferation was measured at the indicated time points in LNCaP cells (**P < 0.01, ***P < 0.001). **D** Transwell assay analyses of the indicated cell lines. **E** Xenograft analyses of DU145 derived tumors with control vector or *ZBTB38* overexpression (**P < 0.01, ***P < 0.001).

To further determine the role of *ZBTB38* in prostate cancer progression, we overexpressed *ZBTB38* in LNCaP, DU145, and PC-3 prostate cancer cell lines (Supplementary Fig. S1A). We first determined the effect of *ZBTB38* on prostate cancer cell proliferation. Results showed that overexpression of *ZBTB38* could significantly inhibit prostate cancer cell proliferation (Fig. 2A–C). Then we did transwell assays to explore the role of *ZBTB38* in prostate cancer cell migration. As shown in Fig. 2D, overexpression of *ZBTB38* in the three cell lines could greatly suppress the migration activities. Finally, results of the xenograft tumor model using DU145 cells also revealed that upregulation of *ZBTB38* expression induced the growth inhibition of tumor in vivo (Fig. 2E).

### *ZBTB38* primarily binds the promoter regions of its target genes

**Fig. 3:**
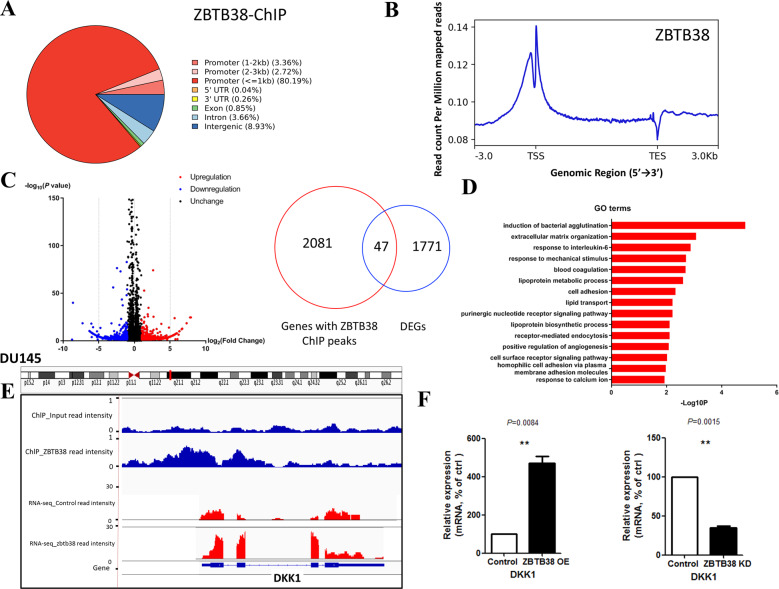
*ZBTB38* binds the promoters and regulates the expression of its target genes in DU145 cell. **A** Genome distribution of HA- *ZBTB38* ChIP-seq peaks in DU145 cells. **B** Signal plot of HA-*ZBTB38* ChIP-seq signals in DU145 cells. **C** Volcano plot for DEGs in *ZBTB38* overexpression DU145 RNA-seq. Right panel: the overlap of ChIP-seq peaks and DEGs. **D** GO analysis (biological process, BP) of downregulated genes after *ZBTB38* overexpression in DU145 cells using the DAVID program. **E** A representative snapshot of *ZBTB38* ChIP-seq and RNA-seq signals located in *DKK1* gene locus. **F** RT-qPCR analyses of the mRNA levels of *DKK1* gene in the DU145 cells with a control vector or *ZBTB38* overexpression or *ZBTB38* knockdown. (**P < 0.01, ***P < 0.001).

Previous studies have determined that *ZBTB38* could serve as a transcription factor implicated in multiple developmental processes [5], we then carried out ChIP-seq assay in order to explore the binding sites of *ZBTB38* in the genome. This experiment yielded a total of 2352 peaks covering 2128 genes, among which 86.27% of the binding sites were in the promoter regions (TSS ±3000 bp). Results of the signal plot analyses showed the binding of *ZBTB38* in the TSS and 5′-end regions of its target genes primarily (Fig. 3A, B).

### Results of functional enrichment analysis of *ZBTB38*-regulated genes

To further analyze the downstream genes and signaling pathways regulated by *ZBTB38*, we performed RNA-seq for the control and ZBTB38-overexpressed DU145 cell lines. After analyzing the results, we identified 1818 genes that were differentially expressed in each group (| log_2_ (fold change) | >1, *P* < 0.05, Fig. 3C). In total, 901 differentially expressed genes (DEGs) were upregulated on *ZBTB38* overexpressing, whereas 917 were downregulated (Supplementary Table S1). Then, we did further analyses combining the results of RNA-seq and ChIP-seq, and found that 47 DEGs were bound by *ZBTB38* at gene body regions or TSS (Fig. 3C), indicating a direct regulation by *ZBTB38*. To further analyze the gene function of DEGs, we did Gene ontology (GO) and KEGG analyses using the upregulated and downregulated DEGs, respectively. Results showed enrichment for gene sets in cell movement and adhesion (Fig. 3D, Supplementary Fig. 1B–D), consistent with our previous phenotypic analyses (Fig. 2).

### *ZBTB38* inhibits prostate cancer cell proliferation and migration via direct upregulation of *DKK1* expression

After validating that *ZBTB38* inhibits the proliferation and migration of prostate cancer cells, we then tried to address the underlining mechanisms. We explored the DEGs that were both directly bound by *ZBTB38* and reported to be implicated in prostate cancer disease progression. Among all eligible candidate genes, *DKK1* is a WNT signaling regulator that plays important role in multiple cancer types. Then we focused on *DKK1*, whose TSS was bound by *ZBTB38* and whose expression was significantly upregulated upon *ZBTB38* overexpression (Fig. 3E). The elevated expression of *DKK1* was also confirmed by qRT-PCR (Fig. 3F).

**Fig. 4:**
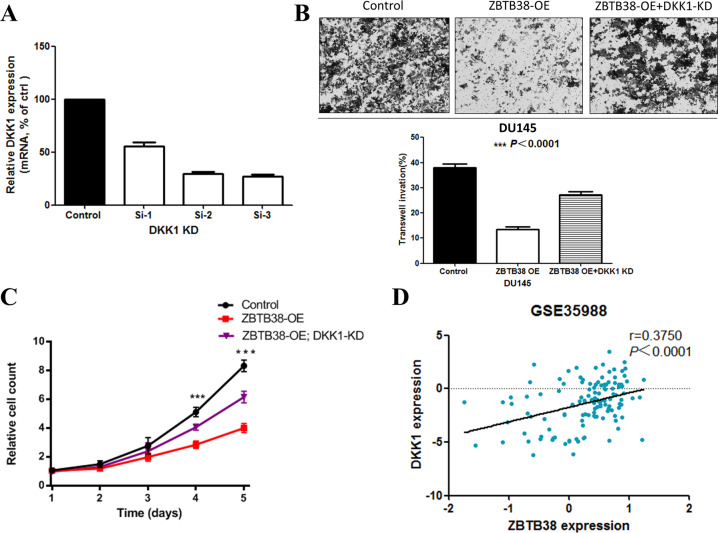
*ZBTB38* suppresses prostate cancer cell proliferation and migration through promoting *DKK1* expression. **A** RT-qPCR showed the mRNA levels of *DKK1* gene in the DU145 cells with a control vector or *DKK1* knockdown SiRNA. **B** *DKK1* knockdown rescued *ZBTB38*-mediated reduced cell migration. One-way ANOVA analysis was used. **C** *DKK1* knockdown rescued *ZBTB38*-mediated reduced cell proliferation (**P < 0.01, ***P < 0.001). **D** Expression correlation analyses of *ZBTB38* and *DKK1* mRNA levels in prostate cancer samples from GSE35988.

To further validate that *DKK1* could mediate the tumor suppressor role of *ZBTB38* in prostate cancer cells, we then knocked down *DKK1* expression in DU145 cells (Fig. 4A). Results of transwell assays showed that the migration ability of *ZBTB38*-overexpressed DU145 was partially restored after *DKK1* suppression (Fig. 4B). Similarly, knocking down *DKK1* also partially mitigated the inhibition of cell proliferation caused by overexpression of *ZBTB38* in DU145 (Fig. 4C). These results were also confirmed in PC-3 prostate cancer cell line (Supplementary Fig. 2A, B). Then we investigated the correlation between *ZBTB38* and *DDK1* expression in prostate cancer samples. We analyzed online publicly available data sets from GSE35988 and found that the mRNA expression level of *ZBTB38* was positively correlated with *DDK1* (Fig. 4D). All these data above indicate that upregulation of *DKK1* is one of the potential mechanisms that mediated the tumor-suppressing function of *ZBTB38*.

### *PRKDC* could interact with *ZBTB38* and inhibit the upregulation of *DKK1* expression

**Fig. 5:**
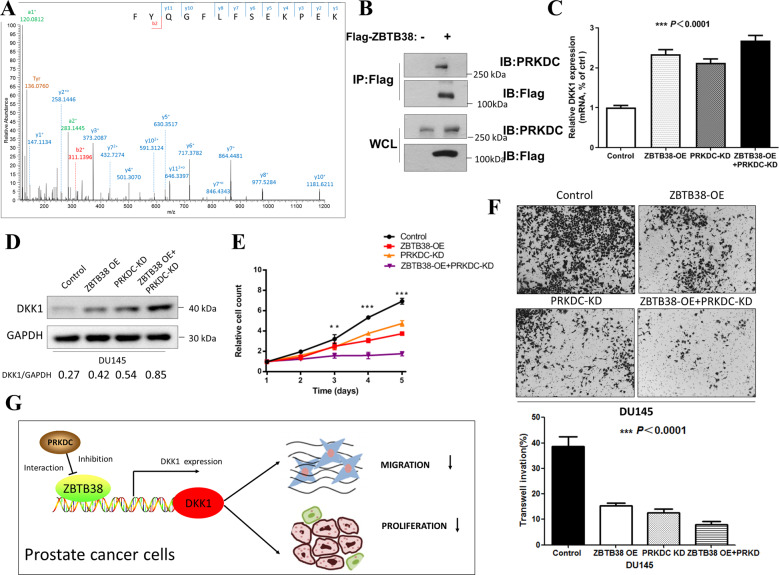
*PRKDC* interacts with *ZBTB38* and inhibits the function of *ZBTB38*. **A** Mass spectrometry identification of a higher-energy collisional dissociation (HCD) MS/MS spectrum was recorded on the [M + 3H]3+ ion at m/z 540.94 of the human prKDC peptide FYQGFLFSEKPEK. Predicted a-, b-, and y-type ions (not including all) are listed above and below the peptide sequence, respectively. The cycle symbol means the neutral loss of water. **B** Co-IP analysis of the interaction between *ZBTB38* and *PRKDC*. **C** *PRKDC* knockdown induced *DKK1* upregulation in mRNA expression level. One-way ANOVA analysis was used. **D** *PRKDC* knockdown induced upregulation of *DKK1* protein. **E** *PRKDC* knockdown reduced *ZBTB38*-mediated proliferation in DU145 cells (**P < 0.01, ***P < 0.001). **F** *PRKDC* knockdown reduced *ZBTB38*-mediated migration in DU145 cells. One-way ANOVA analysis was used. (*ZBTB38* OE+*PRKD* means *ZBTB38* oversexpression with *PDKDC* knockdown in DU145 cells) **G** Working model depicting the mechanism of *ZBTB38*/ *PRKDC*-mediated *DKK1* promotion in prostate cancer.

To explore deeply how *ZBTB38* might regulate *DKK1* expression, we performed Flag immunoprecipitation in DU145 cells with Flag-*ZBTB38* expression followed by mass spectrometry analyses to identify interacting proteins of *ZBTB38* (Supplementary Table S2). Among all possible proteins, we focused on *PRKDC* (Fig. 5A). *PRKDC* (Protein Kinase, DNA-Activated, Catalytic Polypeptide) is a nuclear protein serine/threonine kinase that is a molecular sensor of DNA damage, which has been shown to play important roles in the disease process of a variety of tumors, including breast cancer, colorectal cancer, and prostate cancer [18–20]. Particularly, a previous study confirmed that *PRKDC* is an independent prognostic factor in patients with prostate cancer and downregulation of *PRKDC* inhibited prostate cancer growth [19]. In the co-immunoprecipitation (co-IP) assay, we also confirmed the interaction between Flag-*ZBTB38* and *PRKDC* (Fig. 5B). Importantly, we found that *PRKDC* could inhibit the tumor-suppressive function of *ZBTB38*, as *PRKDC* knockdown (Supplementary Fig. 2C) resulted in the increase of *DKK1* expression and enhanced the tumor-suppressive function of *ZBTB38* (Fig. 5C–F). Moreover, we assessed the expression levels of *ZBTB38* in *PRKDC* knockdown DU145 cells to see whether *PRKDC* knockdown produced lower levels of *ZBTB38* which could lead to the results above. As shown in Supplementary Fig. 2C, *PRKDC* knockdown did not influence *ZBTB38* expression, thus excluding this possibility. Taken together, these findings uncovered the tumor-suppressive roles of *ZBTB38* to suppress cell proliferation and migration in prostate cancer cells. We identified *DKK1* as the direct downstream of *ZBTB38*, which could mediate the tumor-suppressing function. *PRKDC* could interact with *ZBTB38* and potentially be involved in the regulation of *DKK1* expression by *ZBTB38* (Fig. 5G).

## Discussion

Prostate cancer is one of the most common malignant tumors in males and ranks sixth in cancer-related deaths [1]. Among all the cancer types, metastatic prostate cancer and the development of castration-resistant prostate cancer (CRPC) are the major catalysts of mortality in prostate cancer patients [21]. However, the molecular biological mechanisms of the progression of prostate cancer are still not clear, leading to poor prevention and treatment of such aggressive prostate cancer. Therefore, an in-depth study of the molecular mechanisms of prostate cancer disease progression will help find new drug targets and intervention strategies.

Here in this study, we focused on *ZBTB38* and investigated its role in prostate cancer progression. We found that *ZBTB38* expresses much lower in prostate cancer tissues, and its expression is reversely correlated with prostate cancer disease progression. Overexpression of *ZBTB38* could repress the proliferation and migration of prostate cancer cells via direct promotion of *DKK1* expression. Mechanistically, our results indicated that *PRKDC* is an important interacting protein of *ZBTB38*, which could repress the tumor-suppressive function of *ZBTB38*. Knocking down *PRKDC* could significantly promote *DKK1* expression to suppress the malignant progression of prostate cancer cells. These findings are of importance since the molecular pathogenesis of prostate cancer is still poorly known, the identification of *ZBTB38* as a novel tumor suppressor might provide novel possible targets for therapeutics.

*ZBTB38* is a new member of the ZBTB family located on chromosome 3q23 with eight exons. Although previous research has revealed the tumor-suppressive role of *ZBTB38* in prostate cancer [7], however, the mechanisms are still unclear. Besides confirming previous results, our results also showed the binding affinity of *ZBTB38* using ChIP-seq analysis and identified *DKK1* as the direct downstream of *ZBTB38*.expression in mediating its tumor-suppressive role. *DKK1* could inhibit β-catenin-dependent *WNT* signaling by inhibiting *LRP5/6* interaction with *WNT*, and play a role in embryonic development and bone formation in adults [8–10]. Our results suggest the novel role of *DKK1* in prostate cancer, which could serve as the direct downstream of *ZBTB38* in regulating prostate cancer cells proliferation and migration. However, the downstream genes and signaling pathways of *DKK1* in prostate cancer are still unknown, which are also quite intriguing issues. It might be *WNT* pathway or some other novel mechanisms. In the future, we will focus on the role of *DKK1* in prostate cancer, which could help us understand the disease better.

Our study for the first time determined the role of *PRKDC* as an interacting protein associated with the tumor suppressor *ZBTB38* to promote *DKK1* expression in prostate cancer. We used IP and co-IP assays to confirm the interaction between *ZBTB38* and *PRKDC*. In vitro assay indicated that *PRKDC* knockdown resulted in an increase of *DKK1* expression and enhanced the tumor-suppressive function of *ZBTB38*, which suggested that *PRKDC* could inhibit the tumor-suppressive function of *ZBTB38*. The interaction of *PRKDC* and *ZBTB38* is quite interesting. *PRKDC* might phosphorylate some critical sites of *ZBTB38*, and we will focus on the concrete mechanisms in the future.

Nowadays, we still have no effective treatment strategies for patients with advanced prostate cancer (including CRPC and mCRPC). One of the critical reasons is that the mechanisms of how CRPC and mCRPC develop are still not quite clear. Our results indicated the tumor-suppressing role of *ZBTB38* in both AR-positive prostate cancer and CRPC cells. It is possible that *ZBTB38* also contributes to the development of advanced prostate cancer. In the next step, we will also focus on the potential function of *ZBTB38* in this aspect.

Our study contained limitations. In our study, we make an investigation that *ZBTB38* could interact with *PRKDC* to promote *DKK1* expression to suppress prostate cancer cell proliferation and migration. The possible molecular mechanisms of how *ZBTB38* interacts with *PRKDC*, and how the protein complex functions are still not clear and need further study. Moreover, the entire signaling pathways downstream of *ZBTB38-DKK1* still need to uncover in the future.

## Conclusions

Taken together, our findings in this study uncovered the tumor-suppressive roles of *ZBTB38* to suppress cell proliferation and migration by upregulating *DKK1* expression. We also identified that *PRKDC* could interact with *ZBTB38* and regulate its tumor-suppressive roles in prostate cancer.

## Materials and methods

### Cell culture

We obtained LNCaP (*AR* positive), PC-3 (*AR* negative), and DU145 (*AR* negative) cells from ATCC (Bethesda, USA). All cells were tested for mycoplasma contamination and maintained in Roswell Park Memorial Institute (RPMI)-1640 supplemented with 10% fetal bovine serum and antibiotics (0.1 mg/ml streptomycin and 100 units/ml penicillin) as previously [22]. All cell lines used in our study were authenticated by STR profiling and tested for mycoplasma contamination

### Constructs and construction of stable cell lines

We used pPB-CAG-EBNXN vector (Sanger Institute) to deal with constructs and pPB-CAG-ires-Pac was generated as previously described [22, 23]. We ligated full-length *ZBTB38*, *DKK1*, and *PRKDC* into the multiple cloning sites of pPB-CAG-ires-Pac to generate pPB-CAG-*ZBTB38*-ires-Pac, pPB-CAG-*DKK1*-ires-Pac, and pPB-CAG-*PRKDC*-ires-Pac, respectively. Control, *ZBTB38*, *PRKDC*, or *DKK1* overexpression stable cells were obtained as previously described and all stable cell lines were selected and identified by western blotting [22].

### siRNA transfection

In this study, we knocked down gene expression using the individual set of three siRNAs (Shanghai Biotend Biotechnology Co., Ltd, China) against the target genes. The most effective single siRNAs were used for further experiments. Then, 3 × 10^5^ cells per well were subjected to reverse transfection with 20 nM siRNA (Biotend, China) using Lipofectamine 3000 transfection reagent (Invitrogen), following the manufacturer’s instructions for siRNA transfection in six-well plates. The siRNA sequences are shown in Supplementary Table S3.

### Antibodies and immunoblotting

In western blotting, cells were lysed in 1× SDS loading buffer (50 mM Tris-HCl pH6.8, 10% glycerol, 2% SDS, 0.05% bromophenol blue, and 1% 2-mercaptoethanol). Antibodies were listed as follows: anti-*ZBTB38* antibody (21906-1-AP, Proteintech), anti-*DKK1* (21112-1-AP, Proteintech), anti-*PRKDC* (SC-5282, Santa Cruz), anti-HA (51064-2-AP, Proteintech), anti-FLAG (20543-1-AP, Proteintech), anti-*GAPDH* (10494-1-AP, Proteintech), and anti-*TUBULIN* (ab134185, Abcam).

We did immunoblot as previously described [22]. In brief, all proteins were separated by SDS–PAGE and were transferred to polyvinylidene difluoride membranes (Millipore). Horseradish peroxidase-labeled secondary antibodies and an enhanced chemiluminescence system were used for signal detection. Protein was visualized using ChemiDoc XRS chemiluminescence detection and imaging system (Bio-Rad Laboratories) or KODAK film machine.

### Tissue microarrays (TMAs) and IHC

TMAs were constructed using 94 benign prostate tissues and 94 prostate cancer tissues, and IHC was performed as described elsewhere [24]. In brief, slides were deparaffinized and heated in citrate buffer pH6 for antigenic retrieval. The primary antibody was *ZBTB38* antibody (21906-1-AP, Proteintech, 1/600, 30 min). Immunohistochemistry was performed using the streptavidin-biotin-peroxidase method with diaminobenzidine as the chromogen (KitLSAB, Dakocytomotion, Glostrup, Denmark). Negative controls were obtained after omission of the primary antibody or incubation with an irrelevant antibody.

*ZBTB38* staining was scored by two independent observers (including one pathologist) as described previously [24]. In brief, a positive reaction was scored in four grade categories depending on the intensity of the staining and the percentage of *ZBTB38*-positive cells. The sum of the intensity and percentage scores was used as the final score. The staining pattern was defined as follows: 0, negative; 1–2, weak; 3–4, moderate; and 5–6, strong.

### Cell proliferation assay

Cell proliferation (MTS) assay was performed as described elsewhere [25]. In brief, we seeded all cells at 4000 cells/well (0.1 ml) in 96-well plates. All cells were incubated overnight at 37 °C for 5–6 days. At each indicated time point, cells were incubated with 20 μl of CellTiter 96 AQueous One solution reagent MTS (Promega) in 100 μl of RPMI-1640 for one hour. Cell number was then estimated using a microtiter plate reader (Bio-tek).

### In vivo tumor growth assay

In tumor growth assay, we inoculated s.c. (subcutaneous) with 1 × 10^7^ prostate cancer cells in six-week-old male athymic mice. Eight mice were in each group (Control group and *ZBTB38*-OE group) and all mice were sacrificed after 28 days. Tumor growth was monitored weekly, and tumor sizes were measured recorded in length × width^2^ (mm^3^). The Ethics Committee of Nanjing Medical University approved all animal use procedures.

### Cell migration assays

We used Transwell (Corning) system in 24-well tissue culture plates to deal with cell migration assays as described previously [22].

### Real-time RT-PCR assays

We extracted total RNAs from indicated cells in our study using TRIzol reagent (Invitrogen). The extracted RNA was then subjected to reverse transcription with reverse transcriptase (Fermentas). We used the Bio-Rad CFX96 system for quantitative real-time PCR, and normalized the relative gene expression to *GAPDH* as a control. Primer sequences used in the study were shown as follows:

*DKK1*-F: CTCGGTTCTCAATTCCAACG; *DKK1*-R: GCACTCCTCGTCCTCTG;

*GAPDH*-F: GCACCACCAACTGCTTA; *GAPDH*-R: AGTAGAGGCAGGGATGAT

### ChIP-Seq assays

We did ChIP assays as previously described [26]. For HA-*ZBTB38* ChIP, the chromatin was incubated with HA antibody (3724 S, Cell Signaling Technology) overnight at 4 °C, and additionally with pre-washed protein A/G agarose beads (SA032005, Smart-lifesciences) for 1 h. The DNA samples were sequenced by Illumina Novaseq PE150 (Rainbow-genome. Co., Ltd., Shanghai).

### RNA-seq and analysis

We extracted total RNAs from cells in our study using TRIzol reagent (Invitrogen) and genomic DNA was removed using DNase I (TaKara). Then RNA was quantified using the ND-2000 (NanoDrop Technologies). Only high-quality RNA sample (OD260/280 = 1.8~2.2, OD260/230 ≥ 2.0, RIN ≥ 6.5, 28 S:18 S ≥ 1.0, >10 μg) was used to construct sequencing library. The RNA-seq libraries were sequenced on the Illumina HiSeq 4000 sequencing platform at Shanghai Majorbio Bio-pharm Technology Co., Ltd (China).

EdgeR was used for examining the differential expression of RNA-Seq count data as previously described [27, 28]. DEGs were identified with the following criterion: fold change (FC) ≥ 2 or ≤0.5; *P* value <0.05. The Venn calculation result was analyzed using the online tool (http://bioinformatics.psb.ugent.be/webtools/Venn/).

### IP-mass spectrometry

We did all these experiments as described previously [22]. Cells were harvested with EBC lysis buffer supplemented with protease inhibitors (Selleck Chemicals) and phosphatase inhibitors (Selleck Chemicals). For IP, cell lysates were incubated with the FLAG antibody (1–2 µg). Then protein A-Sepharose beads (GE Healthcare) were added to a mixture of cell lysates and antibodies for 1 h. IP complexes were washed five times with NETN buffer. After washing, the IP samples were resolved by SDS–PAGE on a 4–20% polyacrylamide gel (Bio-Rad) and visualized using the Bio-Safe Coomassie Stain (Bio-Rad). The gel containing FLAG-*ZBTB38* complex was excised and all peptide matches were filtered on the basis of mass accuracy, tryptic state (for trypsin), and XCorr, and confirmed by manual inspection. The results of mass spectrometry proteomics data have been provided in Supplementary Table S2 in the supplementary information.

### Publicly available gene expression data sets and clinical data sets

Prostate adenocarcinoma samples from GSE21032 and GSE35988 were used in this study.

### Statistical analyses

All experiments were done in triplicates at least and the results were presented as the average values ± standard error of mean. We did prognosis analysis after surgery using Kaplan–Meier method with log-rank test. Between-group variations in this study were evaluated by the use of the Student’s *t* test, data from more than two groups were analyzed by one-way analysis of variance followed by Dunnett’s test. Spearman’s correlation finished the exploration of the relationships. The variance between the groups that are being statistically compared is similar. A two-sided *P* < 0.05 was thought to have statistical significance. Graphpad 8.0 was used for the analyses.

## Supplementary information

The supplementary figure legends

Sup Figure 1

Sup Figure 2

Supplementary Table s1

Supplementary Table s2

Supplementary Table s3

## Data Availability

All data generated or analyzed during this study are included in this published article and its supplementary information files.
