# Supplementary material for: ZBTB38 suppresses prostate cancer cell proliferation and migration via directly promoting DKK1 expression
Source: Cell Death Dis. 2021 Oct 25;12(11):998. doi: 10.1038/s41419-021-04278-3 (PMC8546125; doi:10.1038/s41419-021-04278-3)
Supplement: Supplementary file 1 — The supplementary figure legends [file 41419_2021_4278_MOESM1_ESM.docx]

Supplementary Figure 1.

A. Western blot analyses of *ZBTB38* expression in the indicated cell lines with overexpression.

B. GO analysis (BP) of upregulated genes after *ZBTB38* overexpression in DU145 cells using the DAVID program.

C. KEGG analysis of upregulated genes after *ZBTB38* overexpression in DU145 cells using the DAVID program.

D. KEGG analysis of downregulated genes after *ZBTB38* overexpression in DU145 cells using the DAVID program.

Supplementary Figure 2.

A. *DKK1* knockdown rescued *ZBTB38*-mediated reduced cell migration in PC-3 cells. One way ANOVA analysis was used.

B. *DKK1* knockdown rescued *ZBTB38*-mediated reduced cell proliferation in PC-3 cells.

C. *ZBTB38* expression level after *PRKDC* knockdown in DU145.

Supplementary Table s1 List of upregulated and downregulated genes in RNA-seq.

Supplementary Table s2 List of *ZBTB38* interacting proteins identified by mass spectrometry.

Supplementary Table s3 Sequences of siRNA.
