# Supplementary figures and images for: ZBTB38 suppresses prostate cancer cell proliferation and migration via directly promoting DKK1 expression
Source: Cell Death Dis. 2021 Oct 25;12(11):998. doi: 10.1038/s41419-021-04278-3 (PMC8546125; doi:10.1038/s41419-021-04278-3)

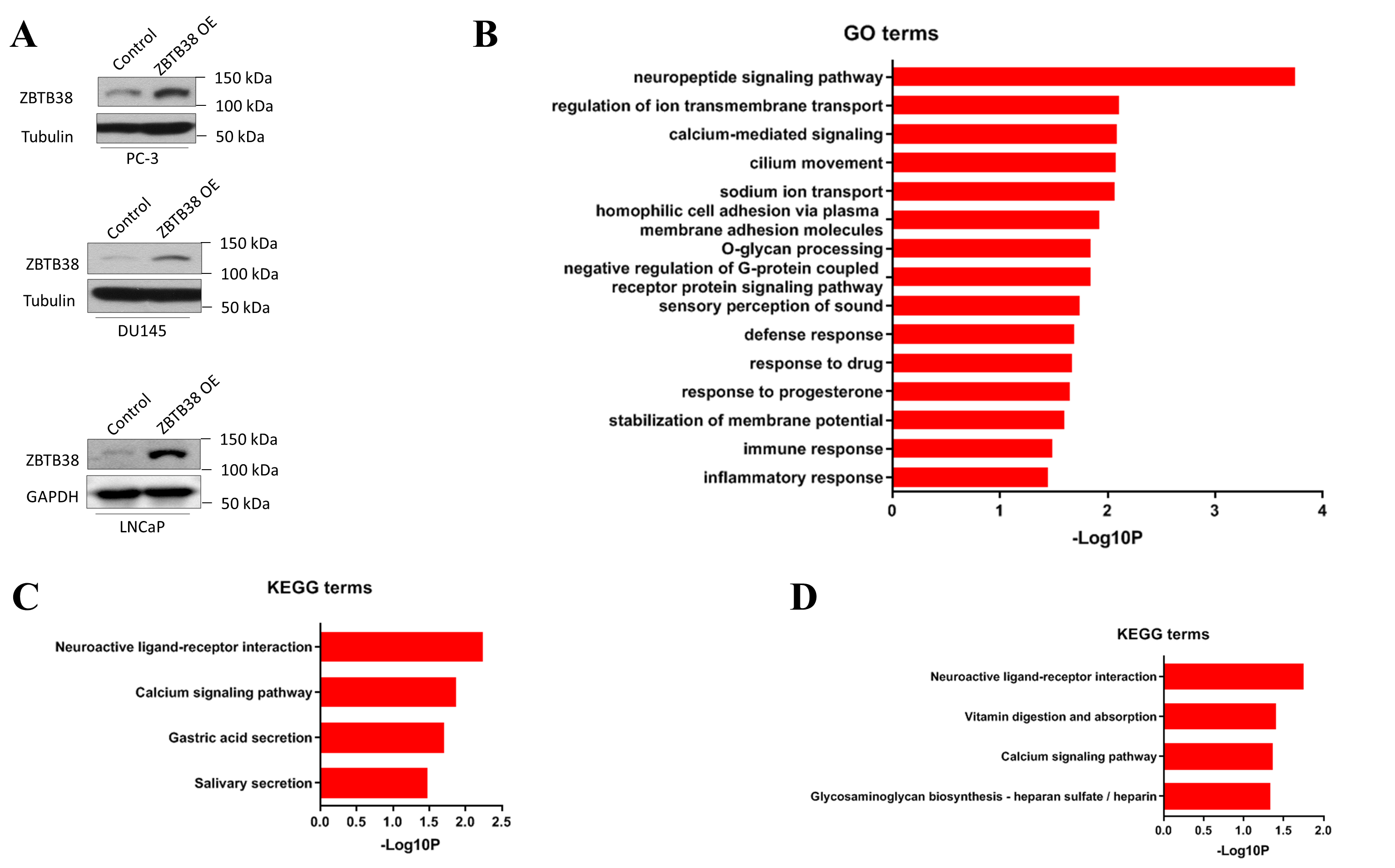

Supplement: Supplementary file 2 — Sup Figure 1 [file 41419_2021_4278_MOESM2_ESM.tif]

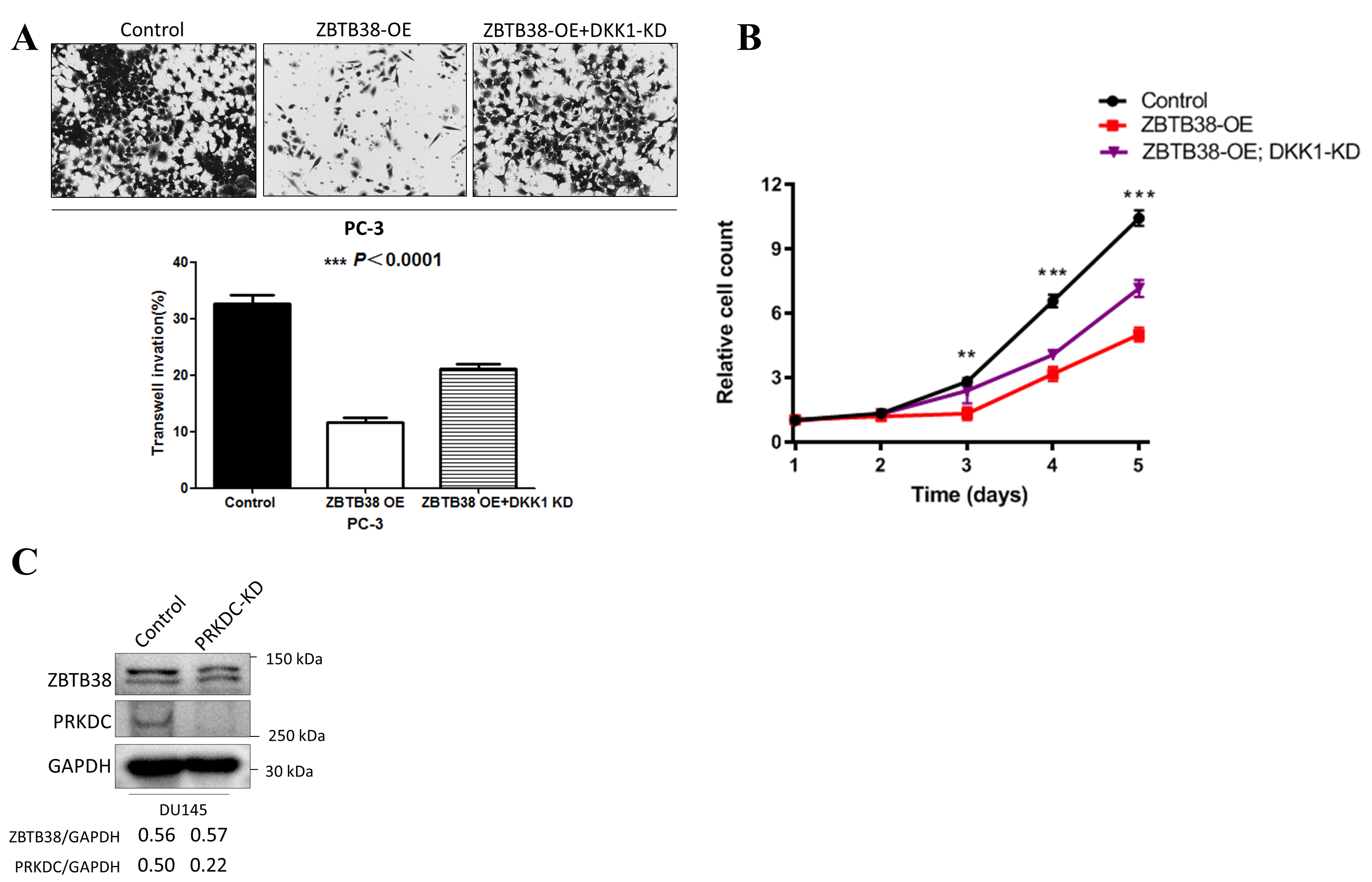

Supplement: Supplementary file 3 — Sup Figure 2 [file 41419_2021_4278_MOESM3_ESM.tif]
